# Metabolic dysfunction associated fatty liver disease in healthy weight individuals

**DOI:** 10.1007/s12072-024-10662-w

**Published:** 2024-07-25

**Authors:** Nahum Méndez-Sánchez, Willem Pieter Brouwer, Frank Lammert, Yusuf Yilmaz

**Affiliations:** 1 Liver Research Unit, Medica Sur Clinic and Foundation, Mexico City, Mexico; 2 Faculty of Medicine, National Autonomous University of Mexico, Mexico City, Mexico; 3 Department of Gastroenterology and Hepatology, Erasmus Medical Center, Rotterdam, The Netherlands; 4 Erasmus MC Transplant Institute, Erasmus Medical Center, Rotterdam, The Netherlands; 5 Health Sciences, Hannover Medical School, Hannover, Germany; 6 Department of Gastroenterology, School of Medicine, Recep Tayyip Erdoğan University, Rize, Turkey

**Keywords:** Insulin resistance, Lipid metabolism, Liver fibrosis, MAFLD, MASLD, Metabolic dysfunction, NAFLD, NASH, Steatotic liver disease

## Abstract

Metabolic dysfunction associated fatty liver disease (MAFLD) is an increasing public health problem, affecting one third of the global population. Contrary to conventional wisdom, MAFLD is not exclusive to obese or overweight individuals. Epidemiological studies have revealed a remarkable prevalence among healthy weight individuals, leading investigations into the genetic, lifestyle, and dietary factors that contribute to the development of MAFLD in this population. This shift in perspective requires reconsideration of preventive strategies, diagnostic criteria and therapeutic approaches tailored to address the unique characteristics of MAFLD healthy weight individuals. It also underscores the importance of widespread awareness and education, within the medical community and among the general population, to promote a more inclusive understanding of liver metabolic disorders. With this review, we aim to provide a comprehensive exploration of MAFLD in healthy weight individuals, encompassing epidemiological, pathophysiological, and clinical aspects.

## Introduction

In parallel to the worldwide obesity pandemic, metabolic dysfunction associated fatty liver disease (MAFLD) is starkly rising in its global prevalence [1, 2]. At this moment, approximately one in three of the worldwide population is affected by this increasingly prevalent liver disease [3]. This leads to an important healthcare burden, with increasing health care costs due to increased utilization of health care resources [4]. Furthermore, the worldwide prevalence of MAFLD is projected to further rise, also because of the increasing prevalence among children and adolescents [3, 4].

Of the MAFLD patients, approximately 25% develop metabolic dysfunction associated steatohepatitis (MASH), which is the more severe form with presence of hepatocyte damage due to inflammation, hepatocyte ballooning and consequently liver fibrosis development and eventually end-stage liver disease with need for liver transplantation. MAFLD is currently the rising indication for liver transplantation listing in the United States [4]. Next to this, MAFLD itself leads to an increased risk of hepatocellular carcinoma (HCC) development [5]. In line with this observation, it is currently observed that the prevalence of HCC due to MAFLD or MASH is also rising [1, 6].

Only recently, MAFLD was referred to as non-alcoholic fatty liver disease (NAFLD). The old”exclusionary” term (i.e. non-alcoholic) reflected something it was not, and it was also believed to be stigmatizing. Therefore, Eslam et al. proposed a nomenclature change and adopted a more inclusive term, which was MAFLD [7]. This new term did not exclude alcohol use, which was viewed as potentially troublesome, and the term fatty still was judged to lead to stigmatization [8]. Therefore, in a recent multi-round, multi-stakeholder Delphi process, a broad term, "Steatotic Liver Disease (SLD)," was adopted [9]. Under the umbrella of SLD, several specific causes are defined, including metabolic dysfunction-associated steatotic liver disease (MASLD), MASH, as well as Metabolic Dysfunction combined with Alcoholic Liver Disease (MetALD). Alcoholic Liver Disease (ALD) holds its own distinct place within the SLD category. Other underlying causes encompass medication-induced SLD, monogenic disorders, and cases where the cause remains unidentified, known as cryptogenic SLD [9].

Although the presence of obesity and metabolic syndrome (MetS), predominantly in the form of insulin resistance (IR), are pivotal in the development of SLD, it is well known that some patients may have MAFLD or MASH without being overweight (defined as a BMI > 25 kg/m^2^ or > 23 kg/m^2^ for Asian populations). This so-called “lean MAFLD” (previously known as lean NAFLD or lean MASLD) patient group pose a complex clinical situation in terms of pathophysiology, risk factors, diagnosis, treatment, prognosis, and economic burden.

By focusing in this review on patients with normal-weight MAFLD, we aim to address the unique challenges and considerations associated with this population. Their potential susceptibility to advanced fibrosis and increased risk of all-cause and cardiovascular mortality [10, 11] further underscore the importance of understanding and treating MAFLD in individuals who do not present the traditional markers of obesity.

Since the recent nomenclature change, NAFLD and MAFLD terms will also be utilized in reference to previous research; it should be noted that MASLD and NAFLD per definition exclude alcohol use, but MAFLD does not (Table 1) [9].

**Table 1:** Progression in definitions of NAFLD, MAFLD and MASLD [6]

NAFLD	Presence of hepatic steatosis (> 5%) in the absence of other etiologies such as alcohol (ab)use, steatogenic drugs or viral hepatitis
MAFLD	Presence of hepatic steatosis (> 5%) in the context of 1 major metabolic criterium (presence of overweight, diabetes mellitus type 2) or 2 minor criteria (hypertension, increased weight circumference [> 102 cm for males, > 88 cm for females], hypertriglyceridemia, high LDL, low HDL levels, prediabetes, or CRP > 2 mg/L) Alcohol use (any level of consumption) is allowed
MASLD	Presence of hepatic steatosis (> 5%) in the context of 1 metabolic criterium (presence of overweight, diabetes mellitus type 2, hypertension, increased weight circumference [> 94 cm for males, > 80 cm for females], hypertriglyceridemia, high LDL, low HDL levels) Alcohol use is allowed up to 20 g/day for females, and 30 g/day for males

## Understanding MAFLD in general

The pathophysiology of MAFLD is complex and multifactorial. There are many different pathways that explain the ultimate fat droplet accumulation in the liver. The most prevailing hypothesis is that this is a multi-hit disorder, comprising of exogenous exposure, genetic predisposition, and the gut microbiome [12]. Combined this may lead to increased visceral adipose tissue, increased insulin resistance and as a result increased influx of free fatty acids due to lipolysis, which may lead to both an altered glucose and lipid metabolism culminating into hepatic steatosis. Due to excess fat accumulation, hepatocyte injury and inflammation may occur due to endoplasmatic reticulum stress and mitochondrial dysfunction, which may further lead to oxidative stress [12]. This leads to further downstream hepatocyte death with necrosis, necroptosis and apoptosis, which further leads to activation of hepatic stellate cells and collagen deposition. This results in fibrosis and eventually cirrhosis formation. Potentially, intestinal dysbiosis may add to this process through production of portal endotoxins [12–14].

## Prevalence of MAFLD in healthy weight individuals

Since there are multiple pathways involved in the development of SLD and steatohepatitis, it is not uncommon to encounter a healthy-weight patient with MAFLD. Previous studies in NAFLD and MAFLD have shown that approximately 10–20% of the total SLD population [15, 16] consists of patients who are of normal weight. In terms of global liver disease prevalence, this still translates to a very significant proportion (~ 6%), which may surpass the global prevalence of chronic hepatitis B (5%), ALD (1.8%) or hepatitis C (2%). [17] The prevalence of lean MAFLD however could vary considerably because of different study populations (i.e. general population, hospital population), different diagnostic methods (biochemistry, ultrasound, transient elastography, MRI or liver biopsy) and possibly due to misclassification (i.e. presence of other factors of steatosis such as steatogenic drugs or non-disclosure of patients drinking behavior). Moreover, lean MAFLD is more often encountered in Asian populations, where normal BMI cut-offs have already been adjusted to lower thresholds (i.e. BMI of 23 kg/m^2^). Because of significant differences in body composition in this population, they may be overrepresented among lean MAFLD populations. This also shows that weight as a standalone measure of metabolic health may be inadequate and should at least be combined with waist circumference, hip circumference, waist to height ratio (WHR) or the combination thereof, to construct a completer picture. Indeed, it has previously been shown that lean MAFLD patients with higher waist circumference (> 102 cm/ > 88 cm for males/females resp.) had a higher risk of (pre)diabetes and liver fibrosis [18, 19].

## Mechanisms of MAFLD in healthy weight individuals

Until now, the pathophysiological mechanisms of MAFLD have been extensively described, with obesity, insulin resistance (IR), and lipotoxicity as key players [20, 21]. Nevertheless, the development of MAFLD in healthy weight individuals represents a complex scenario, including a heterogeneous spectrum of different causes (Table 2), involving a wider variety of underlying factors [22].

**Table 2:** Causes of MAFLD in healthy weight individuals

Genetic predisposition	Genetic variants of PNPLA3, HSD17B13, CYP2B6, MFN2, GLUT9, and GCKR
Dietary factors	Excessive fructose intake, high intake of processed foods, alcohol consumption and high-fat diets
Physical activity	Lack of regular exercise and physical activity
Metabolic	Insulin resistance, visceral adiposity, sarcopenia
Other	Chronic stress, gut dysbiosis

### Exploration of factors leading to MAFLD in individuals with a healthy weight

The study of factors underlying MAFLD in healthy weight individuals has become a critical area of research, challenging the traditional idea that liver disease is related solely to obesity [23]. Various risk factors have been identified as potential triggers for MAFLD in individuals with a healthy weight, encompassing high energy intake, sedentary lifestyle, altered body composition, hormonal disbalances, gut dysbiosis and genetic predisposition [13, 24]. Conventional wisdom often associates healthy weight with a healthy lifestyle, characterized by balanced diet and regular physical activity. Nevertheless, studies have revealed an opposite reality: individuals with healthy weight are often prone to a higher consumption of added sugars, especially fructose, along with a high intake of cholesterol and a reduced consumption of polyunsaturated fatty acids (PUFAs) [25]. The intake of additional sugars, high cholesterol levels, and the deficiency of PUFAs contribute to an energy imbalance in these individuals. When this excess of energy is not used through physical activity, it results in the storage of excess calories in the form of fat [26]. In healthy weight individuals, fat tends to accumulate around internal organs, commonly referred as visceral fat. Visceral fat has been linked to metabolic disturbances, IR and inflammation [27]. A further alteration in body composition in lean individuals associated with MAFLD is sarcopenia, which refers to decreased muscle mass. Sarcopenia has been linked to alterations in metabolic balance, leading to IR and impaired glycemic control due to reduced muscle mass available for insulin-mediated glucose uptake [28]. In addition, individuals with sarcopenia often have limited exercise capacity, which reduces caloric expenditure and further exacerbates IR and metabolic disturbances [29].

### Role of insulin resistance, inflammation, and lipid metabolism in MAFLD development

Previously we have discussed the various factors that can lead to the development of IR and subsequently to the development of MAFLD. IR leads to increased lipolysis and subsequent release of free fatty acids (FFA) from adipose tissue to the liver, which is the main contributor to increased de novo lipogenesis [30]. In parallel, there is an increase in the levels of adipokines and inflammatory cytokines (such as IL-1, IL-6, and TNF-a) promoting IR [14]. Furthermore, visceral adipose tissue contributes to chronic systemic inflammation through increased levels of C-reactive protein (CRP) and proinflammatory cytokines, combined with a reduction in anti-inflammatory cytokines, create a proinflammatory environment that exacerbates metabolic disturbances, leading to the development and progression of MAFLD [31]. The development of MAFLD in individuals with a healthy weight is far from being simple. The intricate interplay of IR, inflammation, and lipid metabolism, influenced by genetic and environmental factors, underpins the pathogenesis of MAFLD in healthy weight individuals.

### Gut microbiota

Gut microbiota has emerged as a pivotal player in various metabolic processes, including the development and progression of MAFLD [32]. Studies have indicated that alterations in the composition and function of gut microbiota can contribute to the pathogenesis of MAFLD, even in healthy-weight individuals [33]. Through the gut-liver axis, a bidirectional relationship has been established between the gut microbiota and the liver. This bidirectional relationship is intricately shaped by various factors, including dietary choices, genetic predispositions and environmental influences [34]. In pathological conditions such as MAFLD, there is a dysfunction of the intestinal epithelial barrier caused mainly by high intake of fats, carbohydrates and food additives [35, 36]. This dysfunction leads to increased intestinal permeability, which facilitates the arrival of pathogen-associated molecular patterns (PAMPs) from microorganisms and metabolites derived from interactions between the intestinal microbiota and its substrates, to the liver via the portal vein [16]. This process triggers a proinflammatory state, promoting the progression of MAFLD to steatohepatitis and hepatic fibrosis [37, 38].

Metabolic endotoxemia, characterized by persistent low-grade systemic inflammation primarily caused by elevated lipopolysaccharide (LPS) levels, further complicates the scenario [39]. The activation of toll-like receptor-4 (TLR4) by LPS triggers the production of numerous pro-inflammatory cytokines. Recent research has uncovered the anti-inflammatory potential of bile acids, demonstrating their ability to suppress inflammation effectively [40, 41]. Nevertheless, in the context of MAFLD, disruptions in bile acid signaling have been observed. Altered bile acid composition, impaired receptor activation, and disturbances in the bile acid pool dynamics contribute to the dysregulation of metabolic processes [42, 43]. In a study by Alharti et al., it was observed that macrophages in healthy-weight individuals with MAFLD exhibited an excessive production of inflammatory cytokines when activated by toll-like receptor (TLR) ligands compared to their healthy counterparts. This heightened response was attributed to alterations in the epigenome of lean MAFLD macrophages, suppressing bile acid signaling and promoting inflammation [44].

### Genetic predisposition

The development of MAFLD in healthy weight individuals cannot be solely attributed to metabolic or environmental factors. Genetic predisposition plays a significant role, influencing how the body handles lipids, responds to insulin, and manages inflammation [45]. In the last few decades, genome-wide association studies, followed by exome-wide analyses, have led to the identification of genetic risk variants and key pathways as drivers of MAFLD, underlying the trajectories from fat accumulation to fibrosis, cirrhosis, and cancer over time in patients with MAFLD [46]. In patients with non-obese NAFLD, the patatin like phospholipase domain containing 3 (PNPLA3) p.I148M allele is more frequent than in other MAFLD patients [47–49] and independently associated with both NASH and fibrosis stage [18]. The mutant PNPLA3 enzyme interacts with α/β-hydrolase-domain-containing 5 (ABDH5), which is causative in Chanarin-Dorfman syndrome (a rare multisystem neutral lipid-storage disease), leading to sequestration of ABDH5, decreased lipolysis, and larger lipid droplets [50]. A study with 63 liver biopsies from healthy weight patients with MAFLD recruited German tertiary referral centers confirmed that the frequency of the common PNPLA3 p.I148A allele (75%) was significantly higher as compared to the other MAFLD patients (55%) or controls (40%), and the risk allele increased the risk of developing MAFLD threefold. According to the population attributable fraction (PAF), up to 50% of MAFLD cases could be eliminated if the PNPLA3 variant was absent [46]. The landscape of genetic predisposition to MAFLD in healthy-weight individuals extends beyond PNPLA3 to encompass several other notable genetic variants. These genetic factors play crucial roles in influencing the susceptibility and progression of MAFLD in individuals with normal body weight. Among the key genetic variants associated with MAFLD in this context are TM6SF2, MBOAT7, HSD17B13, CYP2B6, MFN2, GLUT9, and GCKR polymorphisms. These genetic factors play diverse roles in lipid metabolism, inflammation, glucose regulation, oxidative stress and IR collectively contributing to the intricate pathogenesis of MAFLD in healthy-weight individuals [51–55]. Genetic links with specific ethnic groups can contribute to the differences in how disease is spread, and their severity observed among different populations [23].

## Health implications and complications

Despite appearing healthy on the outside, individuals with healthy weight and MAFLD face significant health implications and complications. Studies have reported that healthy-weight individuals with MAFLD had a higher risk of all-cause and disease-specific mortality than overweight/obese individuals with MAFLD [56].

### Health risks associated with MAFLD in healthy weight individuals

**Fig. 1:**
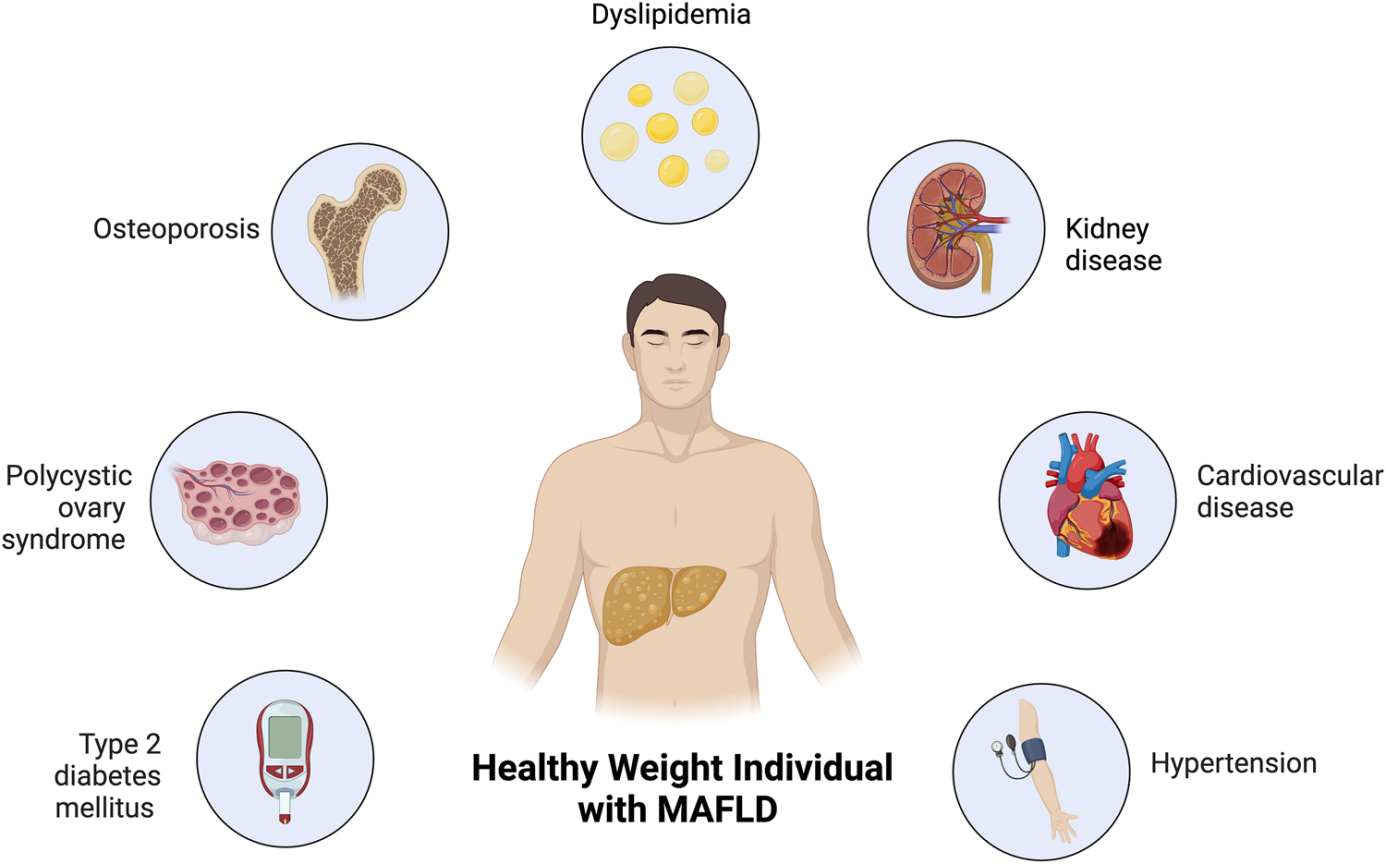
MAFLD associated diseases in Healthy Weight Individuals. This figure illustrates the spectrum of metabolic dysfunction-associated fatty liver disease (MAFLD) related disorders in individuals with a healthy weight. Despite maintaining a healthy body weight, these individuals can still be affected by various metabolic disorders including insulin resistance, cardiovascular complications, and other related health issues

Even in individuals with a healthy weight, MAFLD can lead to severe liver complications. A major complication is liver fibrosis, where excessive fat accumulation triggers inflammation and scarring of liver tissue [57]. It has been demonstrated that healthy weight individuals have more severe liver fibrosis, progression of liver disease as well as higher overall mortality [58, 59]. The impact of MAFLD extends far beyond the liver (Fig. 1). Its association with IR can lead to type 2 diabetes mellitus [60]. Moreover, the chronic inflammation caused by MAFLD can affect the cardiovascular system, raising the risk of heart diseases like hypertension, atherosclerosis, and myocardial infarction [61]. Furthermore, MAFLD can adversely affect the kidneys, impairing their function and potentially leading to chronic kidney disease. The disease also aggravates the risk of metabolic disorders, such as dyslipidemia, in which there is an abnormal amount of lipids in the blood, and polycystic ovary syndrome in female [13]. Finally, sarcopenia in individuals with MAFLD is linked to increased mortality and a heightened risk of significant liver fibrosis [62, 63]. Lifestyle significantly influences this association, with studies indicating that increased levels of physical activity are associated with a decreased likelihood of sarcopenia in individuals with MAFLD [64]. Other health complications associated with MAFLD in healthy-weight individuals are presented in Table 3. These health complications not only decrease quality of life, but also shorten life expectancy if not effectively treated.

**Table 3:** Impact of healthy-weight MAFLD on health outcomes

Health outcome	Impact of healthy-weight MAFLD	References
Prevalence of colorectal adenoma	MAFLD in healthy-weight individuals is associated with the presence of colorectal adenoma, emphasizing the importance of considering colonoscopy examination in patients with MAFLD	[65]
Development of reflux esophagitis	MAFLD in healthy-weight individuals is an independent risk factor for reflux esophagitis, with visceral adiposity emerging as the predominant metabolic risk factor in MAFLD patients	[66]
Recurrence of esophageal squamous cell carcinoma (ESCC)	MAFLD in healthy-weight individuals is independently and directly associated with a higher recurrence rate of ESCC, suggesting MAFLD as a potential marker for identifying individuals at high risk for ESCC recurrence after endoscopic treatment	[67]

## Diagnostic challenges in healthy weight individuals

The available data on histological disease severity in healthy weight patients with MAFLD are contentious, with conflicting results from liver biopsy studies comparing lean and non-lean populations [68–70]. However, from a clinical perspective, there is evidence that lean patients experience similar rates of MAFLD-related comorbidities, such as cardiovascular complications and malignancies, as non-lean patients. Moreover, lean individuals with MAFLD not only face a comparable risk of liver-related mortality as high-risk MAFLD patients with diabetes [71, 72], but they may even exhibit a higher risk of liver-related mortality [73]. Despite these concerning findings, diagnosing MAFLD in lean individuals remains challenging. While current guidelines recommend screening for MAFLD in high-risk categories, such as patients with diabetes mellitus, metabolic syndrome, or individuals who are overweight or obese, they lack tailored and universally accepted screening programs for normal weight subjects [74]. In the subsequent sections, we will explore potential strategies for identifying lean MAFLD and discuss the common hurdles faced in this area.

### Diagnostic tools for MAFLD in patients with normal body mass index

In the context of histopathological examination and imaging modalities, specific recommendations or practice guidelines for identifying *MAFLD* in lean individuals are lacking [74]. This contrasts with obese subjects, where excess subcutaneous fat may require technical modifications for an accurate diagnosis [73]. However, when using blood-based non-invasive diagnostic tools for *MAFLD* screening, adjustments may be necessary to determine the optimal cut-offs for the assessment of hepatic steatosis and fibrosis [75–79]. Furthermore, the diagnostic performances of these non-invasive tests (NITs) have been found to vary for normal weight individuals [79]. Upon analyzing specific NITs, it has been determined that the fatty liver index (FLI) is an effective non-invasive test for lean subjects [75–77]. Previous research has established cut-off values of < 30 for ruling out hepatic steatosis and > 60 for ruling it in, based on data derived from the general population [80]. In a study conducted by Li et al. [75], the performances of eight tests—including FLI, waist circumference-to-height ratio (WHR), visceral adiposity index, aspartate aminotransferase-to-platelet ratio index, hepatic steatosis index, and triglycerides and fasting blood glucose index (TyG)—were compared for predicting MAFLD. FLI and WHR demonstrated superior performance compared to the other tests. The study identified optimal cut-off values of 0.47 and 10 for FLI in lean individuals, and 0.53 and 45 for others [75]. In a separate study by Hsu et al. [77] the authors examined 4000 lean individuals, out of which 740 (19%) were found to have ultrasound-defined NAFLD. FLI showed good performance in detecting lean MAFLD with an area under curve (AUC) of 0.76, sensitivity of 61%, specificity of 79%, and a cut-off point of 15 [77]. Another population-based study in China found that TyG performed excellently in predicting MAFLD in lean patients, with an AUC of 0.92 [81]. The authors also reported that a specific indicator, termed TyG-body mass index (BMI), was the strongest predictor with an AUC of 0.93 [81]. Among the different NITs used for triaging MASLD, the Fibrosis-4 Index (FIB-4) and the Non-alcoholic Fatty Liver Disease Fibrosis Score (NFS) have been recommended for first-line screening. These NITs have shown satisfactory performance for lean individuals [78, 82]. However, there have been inconsistent results regarding the optimal cut-off values for this specific subgroup. A study by Fu et al. [82] included 709 lean individuals with MAFLD and suggested that the current cut-off values for the general population are adequate for lean subjects. Another study conducted in 115 normal weight patients with biopsy-proven MAFLD found similar performance (p = 0.09) between FIB-4 (AUC = 0.81) and NFS (AUC = 0.79), although NFS had lower sensitivity than FIB-4 [82]. Given that the existing evidence primarily relies on single-center studies and indicates potential limitations of FIB-4 and NFS in accurately assessing lean individuals [79], it is crucial to exercise caution when employing NITs in this subgroup. To establish a consensus, further exploration is needed to determine the most suitable non-invasive tools and their respective cut-offs for identifying MAFLD in normal weight subjects. In addition to NITs, laboratory and clinical abnormalities have also been explored in relation to the diagnosis of lean MAFLD. Elevated levels of alanine aminotransferase (ALT), aspartate aminotransferase, triglycerides, uric acid, hemoglobin, and ferritin, along with lower levels of high-density lipoprotein cholesterol, have been associated with the presence and severity of MAFLD in lean individuals [70, 83–85].

Recently, proteomics has emerged as a useful tool for the diagnosis of MAFLD in healthy weight individuals. Jiang et al. observed that the proteomic profile of lean individuals with MAFLD is different from healthy and obese MAFLD individuals. These changes are mainly based on proteins involved in lipid metabolism, the immune and complement systems, and platelet degranulation [86]. Nevertheless, further studies are needed to explore more deeply the use of proteomics in the diagnosis, prognosis, and identification of therapeutic targets in lean MAFLD.

### Risks of underdiagnosis

*MAFLD* is commonly associated with overweight and obesity, leading to a tendency to overlook its occurrence in lean individuals who are typically considered healthy from a metabolic standpoint. However, it is important to note that being lean does not necessarily equate to being metabolically healthy. Lean individuals, defined as those with a BMI of less than 23 kg/m^2^ for Asians and less than 25 kg/m^2^ for other ethnicities [87], can still develop MAFLD. For example, even among lean subjects, an increased fat mass has been associated with the presence of MAFLD [88]. Additionally, lean MAFLD patients with sarcopenia have been found to have higher rates of cardiovascular and liver-related morbidity [89]. MAFLD can also be assessed by transient elastography, allowing simultaneous quantification of liver steatosis and fibrosis [48]. Applying the Asian body mass index cut-off of 25 kg/m^2^, a community-based study in Hong Kong showed that one-fifth of the general non-obese population presented with MAFLD. Therefore, it is crucial to meticulously evaluate all patients, considering their ethnicity and metabolic health, in order to diagnose and prevent complications associated with lean MAFLD.

## Prevention and treatment strategies

Currently, there are no specific guidelines for the prevention and treatment of *MAFLD* in lean subjects. However, the consensus is to prioritize metabolic and liver health. This involves adopting healthy lifestyles, which include dietary modifications and regular physical activity, as these are considered fundamental to managing MAFLD [74]. In the subsequent sections, we will delve into potential strategies for preventing and treating lean MAFLD, drawing from existing data.

### Lifestyle modifications to prevent and treat MAFLD in normal weight individuals

While it is believed that there is a significant overlap between MAFLD as traditionally defined and the new MAFLD definition, current evidence suggests that this may not be the case in the lean population. A study by Ordoñez-Vázquez et al. [90], examined individuals attending regular check-up visits and found that fewer lean patients were classified as having MAFLD compared to NAFLD. Interestingly, lean patients with MAFLD exhibited metabolic unhealthiness characterized by higher BMI, blood glucose, and lipid levels compared to patients with MAFLD [90]. Therefore, it is essential to focus on preserving metabolic health and maintaining a healthy weight to develop effective prevention strategies. A separate study conducted in the USA using data from the third National Health and Nutrition Examination Survey revealed similar results in a general populations sample [91]. In comparison to patients with MAFLD, those with MAFLD were found to be older and exhibited significantly higher rates of hypertension, diabetes mellitus, and insulin resistance. Although MAFLD patients who consumed alcohol showed fewer metabolic disorders, they displayed more severe hepatic damage [91]. To preserve liver health, it is crucial to abstain from alcohol. Since there is no safe threshold for alcohol consumption, maintaining zero intake is recommended [92]. Although alcohol drinking does not represent an exclusion criterion for MAFLD, it is crucial to discourage this habit in all patients diagnosed with this condition. For individuals with advanced fibrosis or cirrhosis, complete abstinence is of utmost importance.

Previous studies have demonstrated that weight loss within the range of 7–8% can effectively lead to the resolution of hepatic fibrosis and steatohepatitis in patients with MAFLD. Moreover, patients who achieved a weight loss of at least 5% over a 52-week period experienced a significant reduction in liver inflammation [93, 94]. Interestingly, even individuals who are already lean can benefit from weight loss efforts targeting a minimum of 5% of their body weight. In a large longitudinal study involving 16,738 adults with MAFLD, Sinn et al. [95] observed a strong correlation between weight reduction and the resolution of fatty liver. This association was particularly prominent in overweight and obese individuals; however, it was also evident in lean participants, with the degree of improvement varying in a dose-dependent manner [95]. Another study from Turkey also demonstrated similar regression rates of hepatic steatosis and fibrosis in both lean and obese individuals [96]. Interestingly, lean patients were more likely to maintain their body weight and liver health in the long-term [97]. Collectively, these findings emphasize the importance of promoting weight loss as a crucial aspect of the management plan for all patients with MAFLD, irrespective of their BMI [98]. Physical activity and diet play pivotal roles in the management and prevention of MAFLD. Adopting a healthy lifestyle, which includes regular physical activity and a well-balanced diet, can have profound effects on metabolic health and liver function [99]. Wang et. al, observed that physical activity and a high-quality diet significantly decreased the risk of MAFLD in healthy-weight and in obese individuals [100]. The Mediterranean diet—characterized by a high intake of omega-3 and monounsaturated fatty acids, along with reduced consumption of refined carbohydrates—is also noteworthy for its positive impact on resolving fatty liver, even without weight loss [98]. In contrast, consuming higher amounts of saturated fatty acids is associated with a higher prevalence and severity of MAFLD [101, 102] In general, avoiding a sedentary lifestyle is crucial for lean patients with MAFLD as they are more likely to face cardiovascular complications rather than liver-related issues [103]. In this regard, both resistance and aerobic trainings may be beneficial.0 A study by Li et al. [104] aimed to investigate the diet and lifestyle characteristics of individuals with MAFLD in China, differentiating between lean and obese patients. The findings revealed that MAFLD patients, regardless of their body weight, demonstrated higher caloric intake, consumed more calorigenic nutrients, grains, potatoes, fruits, and iron, and engaged in extensive overtime work. Moreover, they had shorter sleep durations compared to healthy subjects. Interestingly, normal weight patients exhibited similar dietary and lifestyle patterns to their obese counterparts, underscoring the significance of providing nutritional education and therapeutic guidance specifically tailored for lean MAFLD [104]. Another investigation found that patients diagnosed with biopsy-proven MAFLD, the majority of whom were non-obese, displayed severe liver histology findings, including advanced fibrosis, when they experienced poor sleep quality [105]. This suggests that sleep disruptions may play a crucial role in the progression of liver disease among patients who are not overweight. Collectively, these findings emphasize the importance of addressing sleep disturbances as a potential therapeutic target for individuals with lean MAFLD to prevent further liver damage. For individuals belonging to this patient group, adopting strategies to reduce stress levels, and avoiding overtime work can also yield significant benefits.

### Pharmacological interventions for lean MAFLD

Despite ongoing research efforts into the pharmacological treatment of MAFLD, no therapy has yet received regulatory approval. Particularly, limited data exist on lean MAFLD. A study by Mofidi et al. [106] aimed to evaluate the effectiveness of synbiotics supplementation in MAFLD patients (n = 50) with normal or low BMI. Participants were randomly assigned to a synbiotic supplement or a placebo for 28 weeks, alongside a healthy lifestyle. While both arms experienced reductions in hepatic steatosis and fibrosis, the symbiotic group showed significantly greater improvements. These findings suggest that synbiotic supplementation can improve MAFLD in patients with normal or low BMI by reducing inflammation [106]. Another study by Shinozaki et al. [107] examined non-diabetic MAFLD patients treated with pemafibrate, a selective peroxisome proliferator-activated receptor α modulator, for over six months. The levels of ALT and Mac-2 binding protein glycosylation isomer (M2BPGi) were used to evaluate hepatic inflammation and fibrosis, respectively. The results showed significant improvements in ALT and M2BPGi levels after pemafibrate therapy, regardless of BMI. Specifically, lean patients with MAFLD had a greater reduction in ALT and M2BPGi levels compared to obese MAFLD patients [107]. Considering the existing data, it is crucial to routinely recommend lifestyle modifications and moderate weight loss for individuals with lean MAFLD. Although most research has concentrated on obese individuals, normal weight patients with MAFLD also face similar rates of liver-related complications. Consequently, systematic investigations should be expanded to encompass lean patients, mirroring the approach taken for obese MAFLD cases.

### Follow-up strategies for lean MAFLD

According to available evidence, the follow-up strategy for individuals with lean MAFLD is not yet well-defined. Currently, the approach to surveillance primarily depends on the histological severity of the disease. On the one hand, patients without liver fibrosis and no signs of metabolic deterioration are generally advised to undergo examination every 2–3 years. On the other hand, those with liver fibrosis should have annual screening. For individuals diagnosed with cirrhosis, more frequent surveillance, including hepatocellular carcinoma screening, is recommended every six months. Non-invasive scores and transient elastography are favoured methods for follow-up procedures. Patients at high risk for fibrosis progression, such as those with diabetes mellitus, may need to consider a repeated liver biopsy every 5 years [74]. Although data on the natural history of lean MAFLD are limited and inconclusive, emerging evidence suggests that normal weight individuals, despite having a better metabolic profile and liver histology initially, may exhibit similar long-term disease progression as obese patients [58]. Hence, it would be beneficial to apply the same follow-up strategy to the lean population.

## Conclusions

**Fig. 2:**
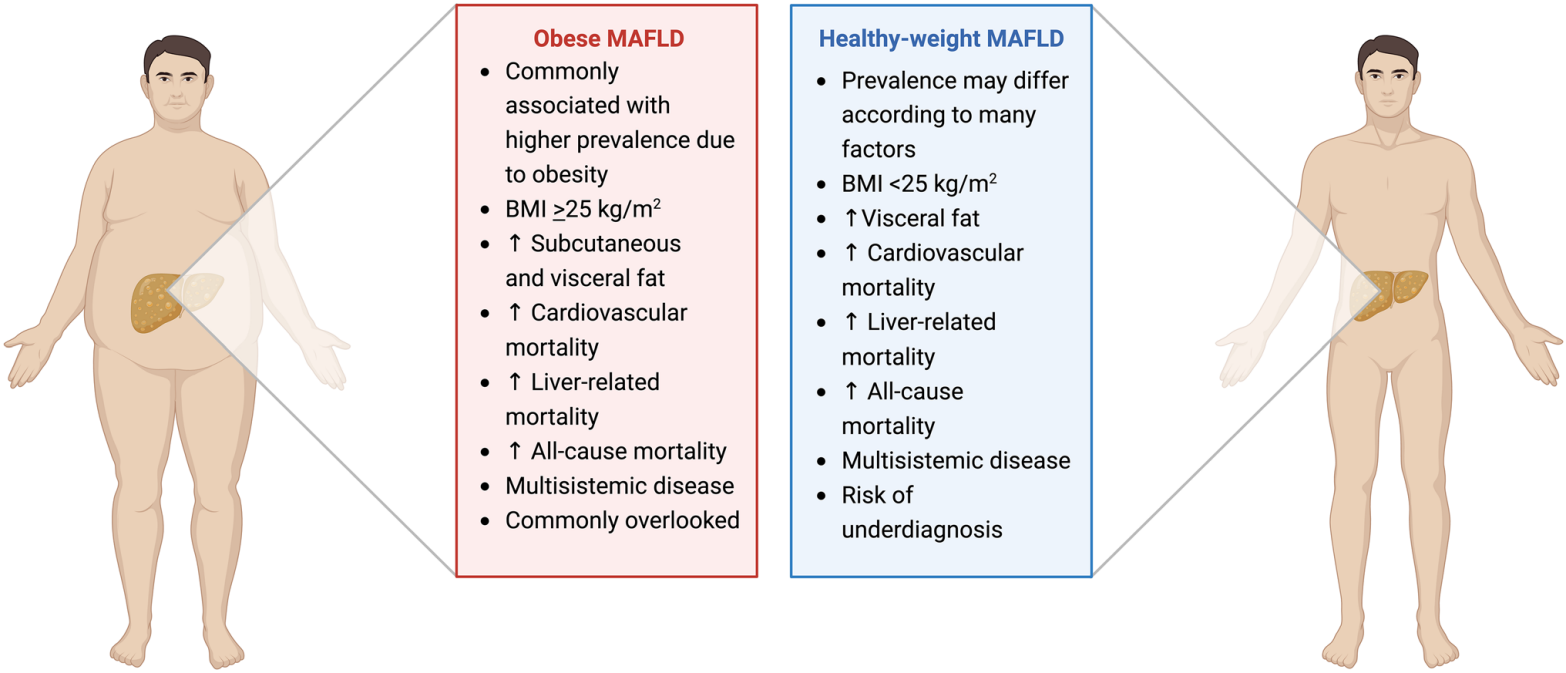
Obese MAFLD vs Healthy-weight MAFLD. Comparative analysis between lean and obese metabolic dysfunction-associated fatty liver disease (MAFLD), highlighting distinctive factors and potential implications associated with these subtypes of metabolic dysfunction-associated fatty liver disease. *BMI* body mass index, *MAFLD* metabolic dysfunction-associated fatty liver disease

The paradigm of MAFLD has evolved beyond its historical associations with obesity, requiring a comprehensive understanding of its development in healthy weight individuals (Fig. 2). The implications and complications associated with MAFLD in healthy weight individuals go beyond the liver, affecting overall health, including increased risk of cardiovascular disease, diabetes, and high mortality. Although health economic models suggest that population-based screening for MAFLD-associated fibrosis might be cost-effective, screening programs must also demonstrate benefit through a reduction in liver-related and/or overall mortality. As the global burden of MAFLD continues to increase, addressing the specific challenges and nuances of MAFLD in healthy weight individuals is critical to advance research, refine diagnostic approaches, and develop targeted interventions to ensure the comprehensive well-being of affected individuals.

## Data Availability

Not applicable.
